# Syphilis Co-Infection Among People Living with HIV in Romania: Epidemiological and Clinical Characteristics in a Single-Center Retrospective Study

**DOI:** 10.3390/pathogens15050465

**Published:** 2026-04-24

**Authors:** Manuela Arbune, Roxana-Elena Bogdan-Goroftei, Alina-Viorica Iancu, Diana-Sabina Radaschin, Florin-Ciprian Bujoreanu, Alin-Laurentiu Tatu, Claudia-Simona Stefan

**Affiliations:** 1 Research Centre in the Medical-Pharmaceutical Field, Medicine and Pharmacy Faculty, “Dunărea de Jos” University of Galati, 800010 Galați, Romaniaalina.iancu@ugal.ro (A.-V.I.); claudia.stefan@ugal.ro (C.-S.S.); 2 “Sf. Cuvioasa Parascheva” Clinical Hospital of Infectious Diseases, 800179 Galați, Romaniaalin.tatu@ugal.ro (A.-L.T.); 3 Multidisciplinary Integrated Center of Dermatological Interface Research MIC-DIR, “Dunărea de Jos” University of Galati, 800010 Galați, Romania; 4 “Sf. Ioan” Emergency Clinical Hospital for Children, 800151 Galați, Romania

**Keywords:** HIV infections, syphilis, coinfection, sexually transmitted diseases, Romania

## Abstract

Syphilis and HIV are sexually transmitted disease (STDs) that interact synergistically. However, data on HIV–syphilis co-infection in Romania remain limited. We conducted a retrospective cohort study at a single Romanian HIV/AIDS Day Clinic, including 439 adult people living with HIV (PLWH) monitored between 2020 and 2025. Demographic, epidemiological, clinical, and laboratory data were collected, including HIV staging and syphilis history. Syphilis co-infection was identified in 81 patients (18.5%), and 61.5% met criteria for AIDS. Viral suppression was achieved in 82.2%, and 78.4% achieved CD4 counts >350 cells/mm^3^. Male sex, urban residence, unmarried status, sexual HIV transmission, genital condyloma, and other STIs were independently associated with syphilis. First episodes of syphilis were predominantly secondary (61%), neurosyphilis was present in 5%, and serofast evolution occurred in 12%, more frequently after reinfection. Among deceased patients, 20.9% had a history of syphilis, but co-infection was not significantly associated with mortality. Nine of 28 patients lost to follow-up had prior syphilis, suggesting a potential impact on retention in care. These findings indicate that HIV–syphilis co-infection is increasingly prevalent in Romania, driven primarily by behavioral factors, and highlight the need for targeted STD screening and prevention strategies among high-risk PLWH.

## 1. Introduction

Infection with human immunodeficiency virus (HIV) and syphilis are two sexually transmitted diseases (STDs) that interact synergistically, as syphilis facilitates HIV transmission, while HIV can alter the natural course syphilis. Both infections are associated with stigma and social discrimination, although the impact is particularly complex in the context of HIV [1].

Syphilis remains a major medical and public health concern, with both sexual and vertical transmission (congenital syphilis). HIV shares similar transmission routes, although blood-borne exposure represents a more significant pathway for HIV than for syphilis [2]. This overlapping epidemiology contributes to the frequent coexistence of the two infections and highlights the need for integrated clinical and public health approaches. Syphilis has long been described as “the great imitator” due to its wide range of clinical manifestations, which can mimic numerous other conditions, while progressing through well-defined stages. The primary stage is characterized by a solitary, nodular ulcer (chancre) often accompanied by regional lymphadenopathy, indicative of bacterial multiplication. Secondary syphilis develops with fever, generalized lymphadenopathy and rash frequently involving palms and soles, corresponding to bacterial dissemination. Following successful treatment and resolution of clinical lesions, serological positivity may persist as early latent syphilis within the first year post-infection or late latent syphilis beyond one year. Tertiary syphilis, developing 20–30 years after initial infection is characterized by gummous lesions and may involve cardiovascular, neurological, ophthalmological and other systemic manifestations [3,4].

HIV infection progresses through well-defined stages determined by the degree of immunodeficiency, defined by the gradual depletion of CD4+ T cells. Primary HIV infection occurs 3–4 weeks post-exposure with nonspecific, self-limiting symptoms [5]. This is followed by a clinically asymptomatic phase, then moderate immunodeficiency and ultimately progression to severe immunodeficiency defined as AIDS [6]. Unlike syphilis, which can be cured with appropriate antibacterial therapy, HIV can only be controlled under antiretroviral therapy, and a definitive cure is not yet available [5,6].

Rates of HIV–syphilis co-infection have increased particularly following the promotion of the “U = U” principle, meaning that undetectable HIV viral load under antiretroviral therapy is untransmissible. Although HIV transmission becomes negligible under viral suppression, reduced risk perception and increased sexual risk behaviors may facilitate the spread of other sexually transmitted infections [7].

Consequently, HIV–syphilis co-infection rates have grown globally over the last two decades, with approximately a threefold rise in the United States (2013–2018), a twofold rise in Canada (2008–2017), a 35% rise in Australia (2013–2017) and a nearly 50% rise in Europe (2009–2018) [8,9,10].

Currently, two epidemiological patterns of syphilis among PLWH can be distinguished: an epidemic driven by transmission in the general population in low-income countries and a re-emergent form predominantly affecting men in high-income countries [9]. It is estimated that 8% of men who have sex with men (MSM) globally are infected with syphilis [10].

The World Health Organization reported elimination of congenital syphilis in some countries, including Cuba, Thailand, Belarus, Armenia, and Republic of Moldova [1], while perinatal HIV transmission has fallen to near zero among women with viral suppression under antiretroviral therapy, highlighting the importance of maintaining the HIV ‘cascade of care’ [8,11,12].

To date, data regarding syphilis among people living with HIV in Romania remain limited. This lack of evidence hinders a comprehensive understanding of the local burden and clinical implications of co-infection.

The objectives of this study were to determine the prevalence of syphilis among PLWH in our center, to compare the clinico-epidemiological characteristics of subgroups defined by syphilis co-infection, and to describe the patterns of syphilis evolution in PLWH.

## 2. Materials and Methods

### 2.1. Study Design

A retrospective, non-interventional cohort study was conducted at a single center: the HIV/AIDS Day Clinic, Clinical Hospital of Infectious Diseases, Galați, Romania. This hospital also includes a dermatology–venereology department authorized for the diagnosis and treatment of sexually transmitted infections (STIs) and is the only medical facility monitoring in the southestern region of Romania, serving a population of over 400,000 inhabitants.

A secondary longitudinal analysis has evaluated the evolutive pattern of syphilis in co-infected prevalent and incident cases during the study period.

### 2.2. Data Collection and Sources

We collected and analyzed demographic data (age, sex, area of residence, education level, marital status), epidemiological data (HIV transmission route), clinical and laboratory data (year of HIV diagnosis, HIV infection stage), and history of other STIs. In the syphilis co-infected subgroup, we collected data on year of syphilis diagnosis, syphilis stage, and clinical and serological evolution (cured, reinfection, serofast).

Data were extracted from the hospital’s clinical registry, selecting cases according to ICD codes: B20–B24 (HIV disease); A51.0–A51.9 (recent syphilis); or A52.0+–A53.0 (late syphilis). Individual medical records of chronic patients who accessed clinic services between 1 January 2020, and 31 December 2025, or until death or loss to follow-up, were also reviewed. Loss to follow-up was defined as no documented clinical visit for more than 12 months [13]. We included cases in which syphilis was diagnosed prior to HIV, as luetic infection may coincide with primary HIV infection, particularly in patients with delayed HIV diagnosis. This approach allows us to consider HIV–syphilis coinfections as synergistic events and to analyze their combined clinical and epidemiological impact.

#### 2.2.1. Inclusion Criteria

Adults aged >18 years with confirmed HIV infection, receiving ART for at least 12 months (2020–2025), were included for the initial analysis of syphilis prevalence and for the comparative evaluation of demographic, epidemiological, and clinical characteristics between patients with and without the infection. Cases were classified as prevalent (documented history or persistent treponemal seroreactivity at enrollment) or incident (new diagnosis during 2020–2025, identified through annual screening or clinical suspicion).

#### 2.2.2. Exclusion Criteria

PLWH without a confirmed serostatus or with less than 12 months of follow-up were excluded to ensure sufficient observation for evaluating the evolution of incident syphilis episodes under treatment. Progression patterns were analyzed exclusively within the affected subgroup.

### 2.3. Case Definitions Used in the Study

HIV infection was diagnosed in symptomatic individuals or through screening of high-risk groups, including pregnant women, tuberculosis patients, blood donors, and individuals with STDs. Diagnosis was confirmed by two reactive ELISA tests followed by Western blotting, according to the national case definition. HIV staging was based on the 1993 CDC classification, including clinical categories A, B, and C, and CD_4_ nadir (>500, 200–500, <200 cells/mm^3^). AIDS was defined as either a CD4 count <200 cells/mm^3^ or the presence of a CDC stage C condition [14,15,16,17,18,19].

The diagnosis of syphilis, specified according to the clinical form of the infection, was established by a dermatologist and was based on clinical and laboratory criteria: quantitative VDRL confirmed by TPHA ± *Treponema* spp. Serological testing was performed using TPHA (Spinreact S.A.U., Sant Esteve de Bas, Girona, Spain), VDRL (DDS Diagnostic S.R.L., Bucharest, Romania), and anti-Treponema IgM ELISA kit (Astra Biotech GmbH, Berlin, Germany), according to the manufacturers’ instructions. IgM, or phase-contrast microscopy was performed in the case of primary chancre lesions, according to the national guidelines [20,21,22,23].

**Figure 1:**
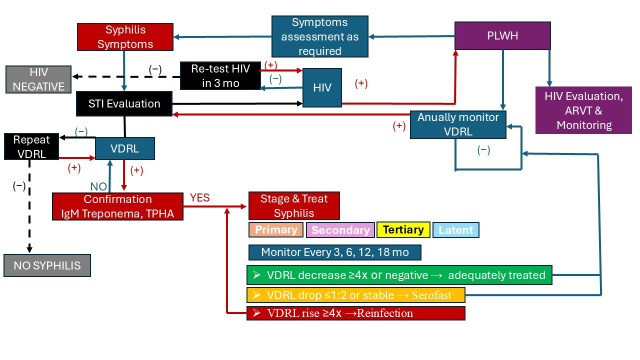
Algorithm of HIV and syphilis management in the Clinical Hospital of Infectious Diseases in Galati (local medical procedures). Legend of arrow colors: red—positive results; blue—follow-up and monitoring workflow; black—negative results.

Syphilis was diagnosed under several clinical scenarios: as a first infection preceding an HIV diagnosis (detected through STI screening or years later) or as a co-infection in individuals already living with HIV (identified through suggestive clinical symptoms or positive VDRL tests during routine monitoring). This diagnostic approach follows the hospital’s standard procedures and is consistent with national guidelines for STI and HIV surveillance (Figure 1).

Syphilis was staged in our cohort according to the case definitions outlined in current clinical guidelines, classifying infections as primary, secondary, tertiary, or latent [17,18,19,20,21,22,23].

### 2.4. Statistical Analysis

Descriptive statistics were used to summarize the study population, with continuous variables, such as age, presented as medians and interquartile ranges (IQR) and categorical variables reported as counts and percentages. Bivariate analyses were performed to assess associations between potential risk factors (e.g., sex, place of residence, education level) and syphilis, with odds ratios (ORs) and 95% confidence intervals (CIs) calculated. The χ^2^ test was applied for categorical variables, with Fisher’s exact test used when expected counts were small, and the Mann–Whitney U test was used to compare non-parametric continuous variables between two groups. Where feasible, multivariate logistic regression (Wald’s test) was conducted to control for potential confounders. Statistical significance was set at *p* < 0.05, and all analyses were performed using XLStat (add-on for Microsoft Excel), version 2024.1.

### 2.5. Ethics Approval

The study was approved by the Ethics Committee of the Clinical Hospital of Infectious Diseases, Galați, no. 40/18 February2026. All patients included in the study provided written informed consent in the monitoring and treatment records for the anonymous statistical processing of personal data for research purposes, in accordance with the hospital’s internal regulations and current legislative standards.

## 3. Results

### 3.1. Dynamics of HIV and Syphilis Co-Infection Cases

Between 2020 and 2025, a total of 439 PLWH were monitored at the clinic and received antiretroviral therapy.

**Figure 2:**
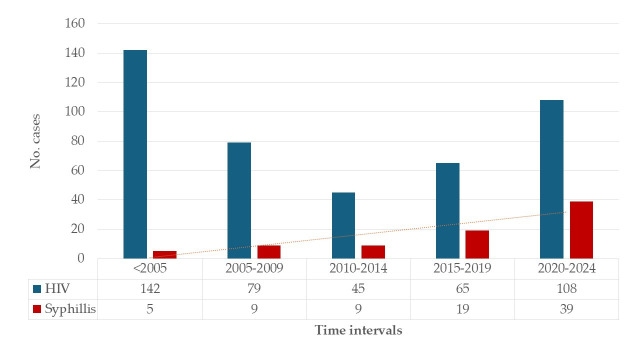
Dynamics of new HIV and syphilis cases. Note: The trendline indicates an increasing trend in syphilis.

The distribution of HIV diagnosis years revealed a heterogeneous cohort structure. A substantial proportion of patients (32.3%) were diagnosed before 2005, reflecting long-term survivors with more than 15 years of infection. At the same time, the 2020–2024 period showed a clear increase in the number of newly diagnosed HIV cases (24.6%) compared to each of the preceding five-year intervals. Syphilis co-infection was identified in 81 patients (18.5%). Syphilis diagnoses increased over time, paralleling the rise in HIV cases (Figure 2).

**Figure 3:**
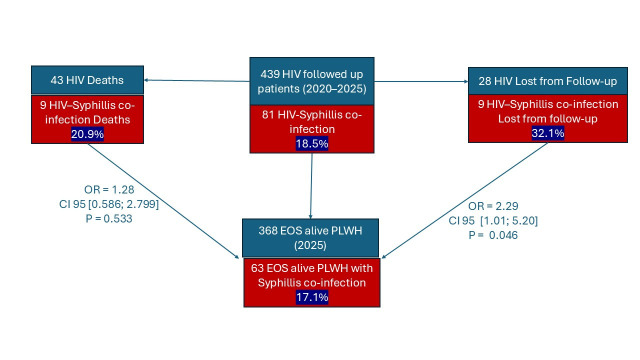
Flow diagram of the HIV cohort stratified by syphilis co-infection and outcomes.

During the study period, 6.3% of patients (28 PLWH) were lost to follow-up, nine of whom had a history of syphilis. Forty-three patients died, corresponding to a mortality rate of 10.46%. Among the deceased cases, 20.9% had a history of syphilis (nine cases), including three cases of neurosyphilis, in which death was attributed to neurovascular complications. Syphilis co-infection was not significantly associated with mortality among PLWH, but it appears to contribute to retention failure in care at our center (Figure 3).

### 3.2. Characteristics of the HIV Study Cohort

All HIV patients were Caucasian. The majority were male (55.13%), from urban areas (52.74%), unmarried (51.71%), and had no more than eight years of formal education (66%). The age of PLWH at HIV diagnosis ranged from 1 to 72 years (median: 24 years), with a mean of 25.85 ± 16.12 years, whereas the age at syphilis diagnosis ranged from 19 to 64 years, with a mean of 35.19 ± 11.22 years (median: 34 years). The distribution of PLWH by route of transmission was as follows: 61% via sexual transmission, 33.7% in a pediatric cohort, 3.63% via perinatal transmission, 0.68% via intravenous drug use, and 1.37% via an unidentified transmission route. The pediatric cohort in Romania includes patients born between 1987 and 1991, infected nosocomially through contaminated blood and instruments. Some cases survived into adulthood [24].

Overall, 61.5% of patients fulfilled the established clinical and/or immunological criteria for AIDS. Among sexually transmitted infections, we also reported: 32.11% HBV, 2.5% HCV, 20% genital warts, and 17.08% other sexually transmitted infections (*Trichomonas*, *Chlamydia trachomatis*, gonorrhea, genital warts, genital herpes).

All patients received antiretroviral therapy according to different treatment regimens based on European AIDS Clinical Society (EACS) guidelines [19]. Viro-immunological outcomes at the end of the study indicated complete and sustained viral suppression in 82.23% of patients and CD4 counts >350 cells/mm^3^ in 78.36% of cases.

### 3.3. Comparative Characteristics of PLWH by Syphilis Co-Infection Status

Of syphilis diagnoses, 28.5% occurred before HIV detection, 31% led to HIV testing, and 39.5% were in patients already known to be HIV-positive.

Among patients who also had syphilis, the median age at HIV diagnosis was 31 years [range: 3–67 years], which was significantly higher compared to those without syphilis, whose median age was 21 years [range: 1–72 years] (Kruskal–Wallis’s test, *p* < 0.001).

Bivariate analysis showed that syphilis was significantly associated with male sex, urban residence, unmarried status, higher education, sexual transmission of HIV, MSM behavior, and a history of other sexually transmitted infections, particularly genital condylomatosis. In contrast, no association was found between syphilis history and AIDS stage, viro-immunological response, or HBV/HCV co-infection (Table 1).

Multivariate logistic regression revealed that male sex (AOR = 3.5, 95% CI: 1.9–6.4), urban residence (AOR = 2.8, 95% CI: 1.5–5.3), higher education (AOR = 1.7, 95% CI: 1.02–2.9) sexual transmission of HIV (AOR = 6.2, 95% CI: 2.9–13.2), MSM behavior (AOR = 5.1, 95% CI: 2.9–9.0), history of genital condyloma (AOR = 2.9, 95% CI: 1.6–5.2) and history of other STIs (AOR = 1.8, 95% CI: 1.01–3.2) were independently associated with syphilis infection. Being married was associated with lower odds of syphilis infection (AOR = 0.45, 95% CI: 0.25–0.82) (Table 2).

**Table 2:** Multivariate logistic regression of factors independently associated with syphilis infection.

Factor	Adjusted OR	95% CI	*p*-Value
Sex (male)	3.5	1.9–6.4	<0.001
Living area (urban)	2.8	1.5–5.3	0.001
Married (yes)	0.45	0.25–0.82	0.008
Education ≥ 12 years	1.7	1.02–2.9	0.041
Sexually HIV transmission	6.2	2.9–13.2	<0.001
MSM behavior	5.1	2.9–9.0	<0.001
Genital condyloma	2.9	1.6–5.2	0.001
Other STI (yes)	1.8	1.01–3.2	0.042

Legend: OR: odds ratio; CI: confidence interval; MSM: men who have sex with men; STI: sexually transmitted infection.

### 3.4. Clinical Course of Syphilis 

**Figure 4:**
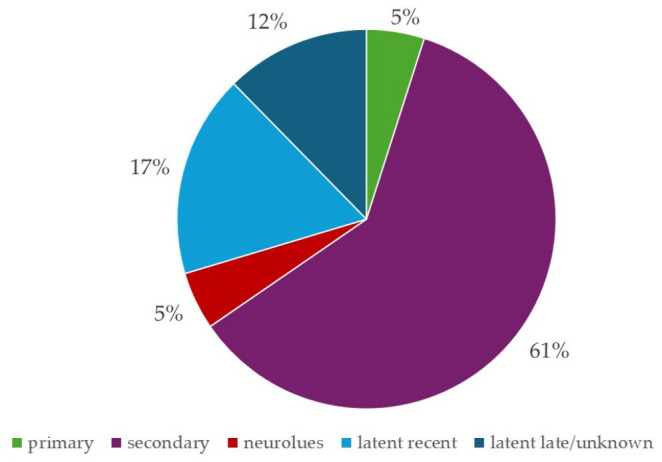
Distribution of Syphilis Stages in PLWH (First Episode Only).

According to medical records, the initial syphilis diagnosis was detected at various stages: 5% primary, 61% secondary, 17% recent latent, and 12% late/unknown duration latent. An additional 5% (four PLWH) of cases were diagnosed as neurosyphilis (Figure 4).

Three MSM were diagnosed with neurosyphilis and subsequently tested HIV-positive. All received treatment; two died from cerebrovascular events, and one progressed to a serofast state after reinfection. The fourth case of neurosyphilis was a woman initially diagnosed with recent latent syphilis who was later diagnosed with HIV. Despite appropriate treatment, she progressively developed manifestations of *Tabes dorsalis* and a thoracic aortic aneurysm, with the particularity of losing treponemal antibodies (TPHA negative) during co-infection. This patient was followed in the clinic for 30 years and died due to rupture of the aortic aneurysm in the context of a COVID-19 episode.

While the cohort of women with HIV–syphilis co-infection was limited, all were of reproductive age. Two cases (12.5%) were identified during pregnancy. Clinical outcomes were favorable following treatment of both mothers and infants.

The first episode of syphilis was cured after appropriate treatment in 92.6% of PLWH (75/81). Six cases, although clinically asymptomatic, maintained low VDRL titers and positive TPHA for more than 12 months after diagnosis and treatment, being classified as serofast syphilis.

Syphilis reinfections were identified in eight PLWH who received appropriate treatment. Among these, four achieved both clinical and serologic cure, while four exhibited a serofast pattern. Comparative analysis indicated that serofast evolution occurred more frequently after reinfection than after the first syphilis episode (Fisher’s exact test, *p* = 0.013; OR = 11.16; 95% CI [2.79–44.68]).

Considering both the first episode and reinfections, serofast evolution was observed in 12% of cases (10 PLWH). According to the guidelines, normal cerebrospinal fluid (CSF) excluded neurosyphilis in 8 of 10 cases, while two asymptomatic patients refused lumbar puncture. No significant correlations were found between serofast evolution and AIDS vs. non-AIDS stage, CD4 count, HIV RNA level, age at HIV diagnosis, or age at syphilis diagnosis. Notably, seven out of eight PLWH with reinfections had undetectable HIV-RNA, although this relationship was not statistically significant.

## 4. Discussion

### 4.1. Epidemiological Characteristics of HIV–Syphilis Co-Infection

Syphilis incidence has increased across the European Union and European Economic area over the past decade, with 41,051 confirmed cases reported in 2023—a 13% rise from 2022 and a two-fold increase since 2014—predominantly among MSM [25]. Although Romania’s absolute notification rates remain lower than the EU/EEA average, national trends similarly show a rise in syphilis cases, particularly during the pandemic and post-pandemic period. This increase is reflected in our cohort, where new infections were observed among people living with HIV, highlighting the continued vulnerability of this population to sexually transmitted infections [25,26].

Our findings demonstrate that syphilis infection among PLWH is independently associated with several socio-demographic and behavioral variables. The persistence of these associations in the adjusted model suggests that individual risk practices play a pivotal role in determining syphilis susceptibility within this population.

Our results align with recent evidence suggesting that syphilis disproportionately affects men, particularly among MSM and HIV-positive individuals [25,26]. This disparity is likely driven by increased engagement in high-risk sexual behaviors and the structure of dense sexual networks within these populations [23,25,27].

The significant association observed between MSM behavior and sexual acquisition of HIV further corroborates contemporary data demonstrating overlapping transmission routes and an intensified burden of syphilis among MSM cohorts [27]. Furthermore, behavioral determinants such as multiple sexual partners and condomless intercourse remain, sustaining transmission cycles [28].

Consistent with the existing literature, our study confirms a strong association between syphilis and a history of other sexually transmitted infections, including genital condyloma. PLWH frequently presents multiple concurrent STDs, reflecting shared behavioral risk factors and synergistic biological mechanisms that facilitate pathogen transmission [27,29].

Additionally, urban residence was associated with higher odds of syphilis infection. This likely reflects increased population density, more interconnected sexual networks, and enhanced access to testing services—factors previously linked to rising STDs rates in recent epidemiological reports [30].

While the association with higher educational attainment has been inconsistently reported in recent studies, our results suggest that individuals with higher education may undergo more frequent screening, potentially leading to higher detection rates rather than a true increase in baseline risk. Finally, the lower prevalence of syphilis observed among married individuals may reflect reduced exposure to high-risk sexual networks, an observation consistent with current epidemiological frameworks [8].

### 4.2. Practical Implications of HIV–Syphilis Co-Infection

The diagnosis and management of syphilis in PLWH continue to present clinical challenges. HIV co-infection is known to influence serologic testing performance, potentially leading to false-positive or false-negative results and complicating accurate staging. In addition, immune dysregulation associated with HIV infection may alter the expected serologic dynamics following treatment, with delayed or suboptimal declines in non-treponemal titers reported in some patients [31,32].

The impacts of syphilis on HIV viro-immunological parameters, including HIV-RNA levels and declines in CD4+ T lymphocyte counts, remain less clear [32]. In our study, no significant associations were observed between syphilis coinfection and key markers of HIV disease progression, including CD4+ T-cell count and HIV viral load, consistent with previous reports in patients receiving suppressive antiretroviral therapy [33,34,35]. Similarly, Huang et al. reported that syphilis infection did not influence immunodeficiency progression in HIV-infected individuals, while Wu et al. found no clinically relevant differences in viro-immunological parameters during long-term antiretroviral treatment [33,34]. In contrast, other studies have described transient immunological alterations during active syphilis infection, particularly temporary reductions in CD4+ T-cell counts, which may reflect immune activation and redistribution of lymphocyte subsets rather than true immunological decline [36,37]. These findings suggest that syphilis may trigger short-lived inflammatory responses, especially in untreated or newly diagnosed infection, but without sustained impairment of immune recovery under effective antiretroviral therapy. Differences across studies may be explained by heterogeneity in study populations, timing of laboratory assessment relative to syphilis diagnosis, and the degree of viral suppression at baseline.

Overall, current evidence, including real-world cohort data [30], indicates that syphilis coinfection does not significantly compromise long-term virological suppression or immunological response in treated HIV-infected individuals, supporting the findings observed in our cohort [32,33,34,35].

### 4.3. Clinical Course of Syphilis in PLWH

In our cohort, 12% of PLWH exhibited a serofast status, defined as the persistence of non-treponemal antibody titers without a fourfold decline despite appropriate therapy. This proportion is consistent with recent studies reporting serofast rates of 11–15% among HIV-infected individuals following treatment [38,39]. Serofast status appears to be more common in PLWH than in HIV-negative populations, likely reflecting both the immunological effects of HIV and factors related to baseline disease stage and initial non-treponemal titers. Although the clinical significance of serofast status remains debated, it underscores the importance of careful longitudinal serological monitoring to differentiate true treatment failure or reinfection from a persistent serological response. These findings reinforce the need for individualized follow-up and highlight that even in the era of effective antiretroviral therapy, serological vigilance remains a critical component of syphilis management in PLWH [38,39].

In this context, our observed reinfection rate of 10%—exclusively among MSM, with seven out of eight cases occurring in individuals with sustained virological suppression under antiretroviral therapy—aligns with the epidemiological profile described in the literature [40,41,42,43,44,45]. The predominance of reinfections among patients with undetectable HIV viral load may support the hypothesis of “treatment optimism” or risk compensation, whereby effective viral suppression reduces perceived transmission risk and may inadvertently decrease adherence to protective sexual behaviors. Such perceptions could facilitate ongoing exposure to sexually transmitted infections, including syphilis, despite effective HIV control.

At the same time, the comparatively lower reinfection rate in our cohort may reflect its specific demographic composition. A substantial proportion of participants originated from the Romanian pediatric HIV cohort, many of whom were infected in early childhood and raised in institutional care settings. This population has faced limited educational opportunities, significant social stigma and discrimination, and barriers to social and professional integration. These structural and psychosocial determinants may shape patterns of partnership formation and sexual behavior. In contrast to cohorts predominantly composed of urban MSM with high partner turnover, a significant proportion of people in the present study appeared to prioritize long-term relationship stability and family formation within traditional partnership frameworks. This reduced exposure to frequent partner turnover—a well-documented driver of syphilis reinfection [46,47]—may partially account for the lower reinfection rate observed in our cohort. Consequently, these findings likely reflect specific socio-behavioral characteristics rather than inherent differences in biological susceptibility [46,47,48].

However, newly diagnosed HIV infections in Romania over the past decade demonstrate a transmission pattern increasingly aligned with that observed in other European countries, with a growing proportion attributable to sexual transmission, particularly among MSM. As the epidemiological profile shifts away from the historical predominance of long-term survivors from the pediatric cohort, future patient populations may exhibit behavioral and reinfection patterns more like those reported in Western Europe, potentially including higher rates of sexually transmitted infections and syphilis reinfection [25].

Furthermore, the high frequency of syphilis reinfections among sexually active PLWH highlights that the risk of infection is more strongly driven by behavioral and exposure-related factors than by the biological status of HIV infection. Our data support this observation, indicating that, despite effective virological suppression, individuals remain vulnerable to sexually transmitted infections. These findings have important clinical implications, emphasizing the need for active monitoring, targeted sexual health education, and preventive interventions within high-risk subgroups to reduce both the incidence and reinfection of syphilis among PLWH.

### 4.4. Limitations

This study has several limitations that should be acknowledged. First, the relatively small number of cases, particularly within stratified subgroups, may have limited the statistical power to detect weaker associations. Second, the extended time span of data collection overlaps with evolving diagnostic and therapeutic guidelines, especially in HIV management, and the exposure of a proportion of patients to multiple antiretroviral regimens may have introduced heterogeneity, affecting the comparability of data across different periods.

Additionally, the partially retrospective design raises the possibility of incomplete or heterogeneous historical documentation, and self-reported behavioral data may be subject to reporting bias. Variability in serological testing strategies over time, including the absence of documented treponemal confirmation in some earlier records, may have led to a potential underestimation of the true lifetime prevalence of syphilis in this cohort.

Furthermore, in deceased cases, the lack of neuropathological examination and immunohistochemical confirmation limits definitive conclusions regarding the underlying mechanism of central nervous system involvement. Although neurosyphilis was excluded in most serofast cases based on normal cerebrospinal fluid findings according to contemporary guidelines, lumbar puncture was declined in a minority of clinically asymptomatic patients, which may represent a source of residual diagnostic uncertainty.

Despite these limitations, the consistency of the observed associations and the integration of clinical and laboratory data strengthen the validity of the findings.

## 5. Conclusions

The prevalence of syphilis among PLWH in our cohort was 18.45%, with an increasing trend observed over the last decade. Syphilis co-infection was significantly associated with male sex, particularly among MSM, urban residence, lower and medium educational levels, and a history of other sexually transmitted infections, especially genital condylomas. In our cohort, syphilis co-infection did not significantly impact HIV disease progression or the virological and immunological response to antiretroviral therapy. HIV infection was associated with a slower serological response following syphilis treatment, with a serofast state documented in 12% of cases. A higher frequency of this condition was observed in patients with syphilis reinfection compared to those experiencing an initial episode. Systematic serological monitoring of syphilis in PLWH remains essential for early diagnosis and timely treatment, contributing to the prevention of late-stage complications.

## Figures and Tables

**Table 1:** Characteristics of PLWH by syphilis co-infection status (2020–2024).

	Categories	%	n1 (Syphilis+)	n2 (Syphilis−)	OR	CI 95%	*p*
Sex	Male	55.13%	65	177	4.15	2.38; 7.23	<0.001
	Female	44.87%	16	181			
Living area	Urban	52.74%	62	169	3.63	2.13; 6.17	<0.001
	Rural	47.26%	19	188			
Married	Yes	48.29%	25	56	0.40	0.24; 0.67	<0.001
	No	51.71%	187	171			
Education	≥12	33.79%	111	246	1.86	1.14; 3.03	0.012
	<12	66.21%	37	44			
Transmittion Pattern *	Ped Cohort	35.57%	6	142	9.02	4.31; 18.89	<0.001
	Sexual	64.42%	74	194			
Sexual behaviour **	MSM	14.88%	34	30	7.69	4.54; 13.04	<0.001
	HS	85.11%	47	319			
Genital Condyloma	Yes	20%	33	55	3.78	2.28; 6.28	<0.001
	No	80%	48	303			
Other STI	Yes	17.08%	30	80	2.04	1.22; 3.39	0.005
	No	82.29%	51	278			
HBV	Yes	32.11%	28	113	1.14	0.68; 1.90	0.601
	No	67.88%	53	245			
HCV	Yes	2.5%	1	10	0.42	0.05; 3.19	0.407
	No	97.49%	80	342			
AIDS	Yes	61.50%	51	219	1.07	0.65; 1.77	0.764
	No	38.49%	30	139			
Deaths (2020–2025)	Yes	10.46%	63	305	1.28	0.58; 2.79	0.533
	No	89.53%	9	34			
RNA-HIV ***	Undetectable	82.23%	71	290	0.60	0.29; 1.21	0.157
	Detectable	17.77%	10	68			
CD4	≥350/mm^3^	78.36%	69	275	1.73	0.90; 3.33	0.098
	<350/mm^3^	21.64%	12	83			

Legend: OR, odds ratio; CI, confidence interval; MSM, men who have sex with men; HS, heterosexual; HBV, hepatitis B virus; HCV, hepatitis C virus; RNA-HIV, human immunodeficiency virus ribonucleic acid; *p*: χ^2^ test or Fisher’s exact test was applied as appropriate; * *416 PLWH *(pediatric + sexually infected); vertical, IVDU, unknown excluded. ** 433 PLWH analyzed; 9 sexually inactive excluded; *** HIV-RNA detection limit 50 c/mL.

## Data Availability

The data are not publicly available due to privacy or ethical restrictions but are available from the corresponding author upon reasonable request.

## Abbreviations

The following abbreviations are used in this manuscript:

AIDS	Acquired Immunodeficiency Syndrome
CDC	Centers For Disease Control
CI	Confidence Interval
COVID-19	Coronavirus-19 Infection Disease
CSF	Cerebrospinal Fluid
EACS	European AIDS Clinical Societies
ELISA	*Enzyme-Linked Immunosorbent Assay *
HBV	Hepatitis B Virus
HCV	Hepatitis C Virus
HIV	Human Immunodeficiency Virus
IDCH-GL	Infectious Diseases Clinical Hospital Galati
ICD	International Classification Diseases
LT	Lymphocyte T
MSM	Men who have Sex with Men
OR	Odds Ratio
PLWH	People Living with HIV
RNA	Ribonucleic Acid
STI	Sexually Transmitted Infections
TPHA	*Treponema pallidum Hemagglutination Assay*
VDRL	*Venereal Disease Research Laboratory*
